# Successful DIEP flap breast reconstruction in a patient with previous urostomy, abdominal liposuction, and caesarean section: a case report overcoming a relative contraindication

**DOI:** 10.1080/23320885.2026.2732461

**Published:** 2026-09-16

**Authors:** Muzammil Arif Din Abdul Jabbar, Thilakshi Subasinghe, Charles M. Malata

**Affiliations:** ^a^ School of Clinical Medicine, University of Cambridge, Cambridge, UK; ^b^ Department of Plastic and Reconstructive Surgery, Cambridge University Hospitals NHS Foundation Trust, Cambridge, UK; ^c^ School of Medicine, Anglia Ruskin University, Essex, UK

**Keywords:** DIEP flap, autologous breast reconstruction, abdominal scars, previous liposuction, urostomy

## Abstract

The deep inferior epigastric perforator (DIEP) flap is the most popular option in autologous breast reconstruction post-mastectomy. However, extensive prior surgical history, especially abdominal, urological and/or pelvic surgery are generally considered as relative contraindications to DIEP reconstruction. We report a case of a 63-year-old who successfully underwent breast reconstruction using a DIEP flap despite previous urological surgery and indwelling suprapubic catheter with significant adjacent scarring in addition to prior abdominal liposuction and caesarean section. The reconstruction was carried out with several intraoperative precautions, including flap harvest incorporating three perforators to optimise perfusion and reduce the risk of flap necrosis. Additionally, the contralateral abdominal hemi-flap was left attached until successful microvascular anastomoses were confirmed. The urostomy with its indwelling suprapubic catheter was isolated during surgery with a transparent waterproof dressing to reduce contamination of the operative field. To our knowledge, this is the first reported case of successful DIEP flap breast reconstruction in a patient with a pre-existing urostomy. This case further supports the notion that a complex abdominal surgical history should not be viewed as an absolute contraindication to DIEP flap reconstruction.

## Introduction

Breast reconstruction following mastectomy is an important step in the holistic treatment of breast cancer for psychological and aesthetic rehabilitation. The choice of reconstruction method depends on patient preference [1,2], medical history, body habitus especially fat distribution [3] and impact on quality of life and subsequent functionality. Autologous tissue reconstruction has shown improved satisfaction outcomes compared to implant-based reconstruction [4], and the DIEP flap is a popular option amongst surgeons and patients. However, in cases where patients have extensive abdominal surgical history, the DIEP flap may be contraindicated or challenging [5]. Here, we report successful DIEP flap breast reconstruction in a patient with extensive abdominal and pelvic surgical history and, for the first time in the literature, a pre-existing urostomy.

## Case report

**Figure 1.**
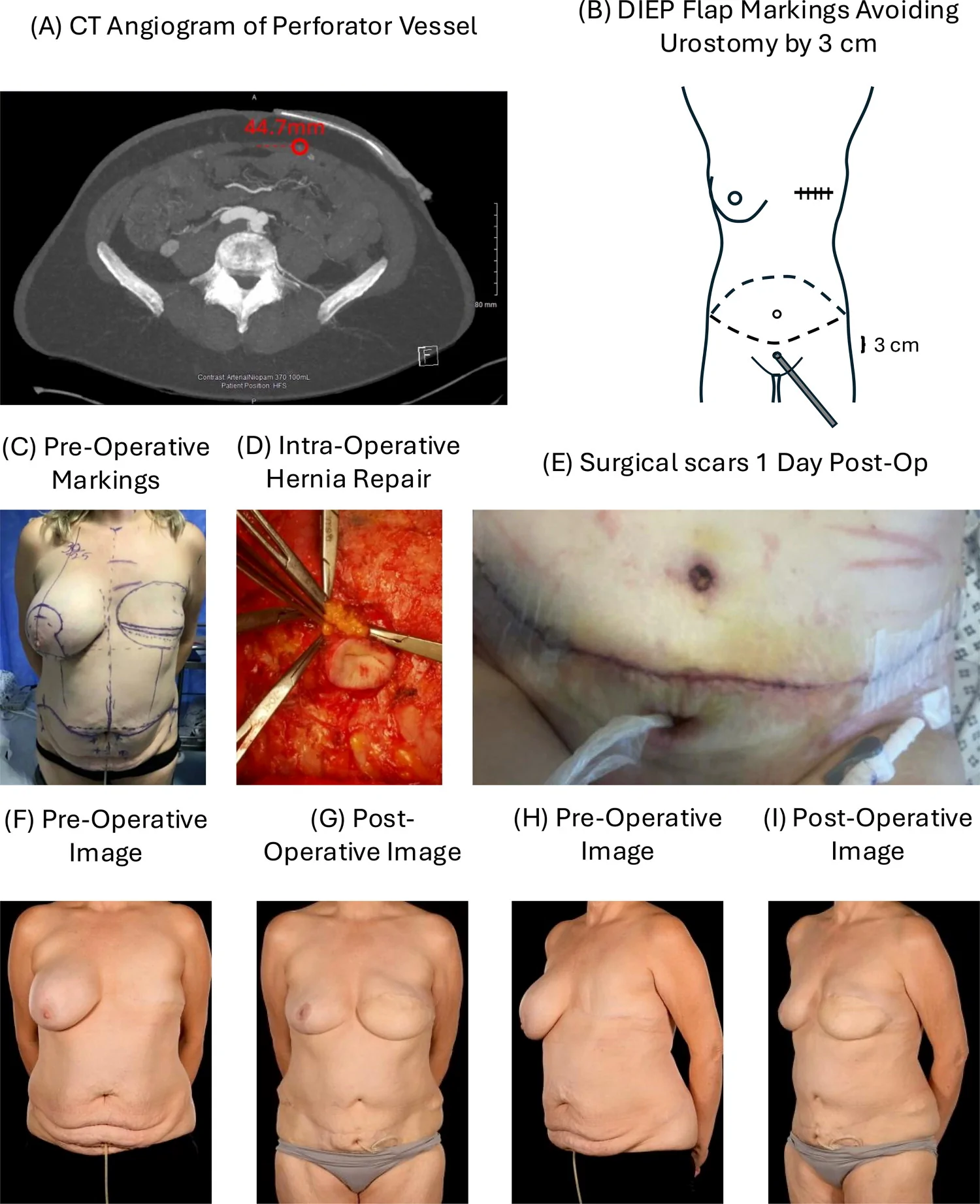
(A) Pre-operative CT Angiogram identified three viable perforators, one of which is shown. (B) DIEP flap markings indicating position of urostomy. Markings avoided urostomy by about three centimetres. (C) Pre-operative appearance of mastectomy deformity and significant size and shape asymmetry of the contralateral breast. Markings indicative DIEP flap harvest location, transfer location and mastopexy markings. (D) Intra-operative incidental hernia repair. (E) Donor site scar, one day post-op with a suprapubic catheter *in situ*. (F, H) Pre-operative images showing a previous left mastectomy and implant-augmented right breast complicated by capsular contracture. There is an adequate abdominal pannus despite the pre-existing scarring. (G, I) Post-operative appearances 16 months later demonstrating the soft supple reconstructed left breast with a recreated inframammary fold. The uplifted and explanted right breast facilitated implant achievement of volume and shape symmetry.

A 63-year-old woman requested delayed breast reconstruction following a mastectomy two years prior for an 80 mm high-grade ductal carcinoma *in situ* with a 3 mm focus of grade 2 invasive carcinoma. She had not received chemotherapy or radiotherapy. She had no other significant medical comorbidities, was not taking any regular medications, and was classified as American Society of Anesthesiologists (ASA) Physical Status II. She has a complex previous surgical history, including caesarean section in her mid-30s, bilateral cosmetic breast implants 22 years prior, liposuction of lower abdomen, thighs, and arms 19 years prior, and supra-pubic catheter insertion two years prior for a complex recalcitrant urethral stricture. She was also concerned about the larger previously augmented breast which had subsequently developed capsular contracture (Figure 1C, F, H).

She was not keen on implant-based reconstruction due to concerns about potential capsular contracture and interference with future breast cancer screening. She also declined both pedicled latissimus dorsi and pedicled or free TRAM flaps, citing the risk of shoulder and/or abdominal wall weakness that could impact her physically demanding job as a plasterer. Additionally, options such as the transverse upper gracilis (TUG) flap and the inferior and superior gluteal artery perforator (IGAP/SGAP) flaps were considered unsuitable, as they would not have provided sufficient volume for breast reconstruction.

Given these factors, reconstruction with a DIEP flap remained the only feasible option, despite her history of abdominal, urological, and gynaecological surgery.

It was further decided with the patient to reduce the volume requirements by a simultaneous contralateral mastopexy and implant removal without replacement to enable use of an appropriately sized hemi-flap. Urological opinion was sought regarding whether to reposition the suprapubic catheter and its tract. It was decided to circumscribe rather than reposition or remove it. The inferior incision for flap harvest was made well above the Pfannenstiel scar and urostomy in order to avoid damaging it and face difficult re-catheterisation of the patient through a very scarred urethra and to avoid leaving a narrow skin bridge which would lead to marginal skin necrosis and inevitable wound breakdown. The DIEP flap design thus followed standard markings; however, the inferior border of the abdominal ellipse was positioned approximately three centimetres above the urostomy, allowing the stoma to remain undisturbed (Figure 1B, C, E). The flap was then elevated, and the abdominal donor site was closed by advancing the upper abdominal flap to the lower abdominal flap without requiring urostomy repositioning or revision.

The procedure was performed with no complications as standard DIEP flap dissection with extra precautions as mentioned above. To reduce infection, the suprapubic catheter was covered with a waterproof opsite dressing which was transparent but occlusive to avoid urine contamination of the operative field. Computerized tomographic angiography (CTA) had identified three viable perforators (Figure 1A), and all three were included in the flap harvest to optimise flap perfusion. The left hemi-flap was raised avoiding the urostomy and trimming the scarring adjacent to it while the right side remained *in situ* until the microvascular anastomoses were successfully completed. The dissection of the perforators was uneventful with no undue scarring around them. The flap weighed 682 g. The internal mammary vessels were exposed in the second intercostal space *via* the senior author’s total rib preservation technique [6] and used as the recipient for the anastomoses. Good flow of artery was confirmed with a handheld doppler and clinically. The entire hemi-flap was adequately perfused as indicated by colour, capillary refill and bleeding from the edges. Contralateral explantation with capsulectomy and mastopexy was then performed. An incidental umbilical hernia was also repaired (Figure 1D). The operation time was 11 h, and estimated blood loss (EBL) 780 ml. This was broadly in line with operating times for similar surgeries at our centre for six months prior (Table 1) and range of EBLs at 234–651 ml. Considering the operative time, daily mobilisation, TED stockings and daily dalteparin was administered as prophylaxis in accordance with European plastic surgery guidelines [7]. No thromboembolic complications occurred. The 11 h was required due to the complexity of the surgery, including the marked abdominal scarring, simultaneous contralateral balancing capsulectomy, breast lift and incidental hernia repair.

**Table 1.** Summary of DIEP flap breast reconstructions performed at the same hospital in the last 6 months.

Age (years)	Procedure	Operative Time
37	Bilateral mastectomy with bilateral DIEP flap reconstruction	17 h
53	Left SSM + SLNB + immediate DIEP reconstruction	9 h
48	Right SSM + SLNB + immediate DIEP reconstruction	10 h
40	Left SSM + SLNB + immediate DIEP reconstruction	10 h
40	Right SSM + SLNB + immediate DIEP reconstruction	10 h
53	Right SSM + SLNB + immediate DIEP reconstruction	9 h
51	Right SSM + axillary clearance + immediate DIEP reconstruction	9 h
37	Delayed left breast reconstruction with contralateral DIEP flap	8 h
53	Right SSM + SLNB + immediate DIEP reconstruction	10 h
67	Left SSM + SLNB + immediate DIEP reconstruction	10 h
56	Delayed left breast reconstruction with free DIEP flap	9 h
71	Delayed left breast reconstruction with DIEP flap	10 h

SSM: skin sparing mastectomy; SLNB: sentinel lymph node biopsy.

The post-operative course was uneventful, with drains removed four days post-op and two units of red blood cells transfused for mild but symptomatic anaemia, although the postoperative haemoglobin was borderline at 83 g/L. The DIEP flap survived completely, and suprapubic catheter has continued to function well (Figure 1E, G, I). There have been no problems in wound healing or any infection of the abdominal wall around or remote from the urostomy after 16 months (Figure 1G, I).

## Discussion

Patients with previous abdominal and pelvic surgeries frequently present for post-mastectomy breast reconstruction and autologous tissue transfer, specifically DIEP flap, may be the only viable procedure in some cases such as the patient herein described. The DIEP flap provided the best aesthetic match for the breast, and the patient had adequate abdominal skin and fat despite her previous abdominal surgeries. The use of pedicled abdominal flaps in such patients raises several concerns, including adhesions due to previous surgeries, unreliable vasculature and poor integrity of the abdominal wall leading to laxity and herniation [3]. Despite these theoretical risks, a meta-analysis previously showed that patients with previous abdominal surgery were no more likely to have post-operative complications after abdominal free flap breast reconstruction, except for an increased likelihood of delayed donor site wound healing [8,9]. This study did, however, exclude liposuction patients. In contrast, the case reported here did not have any healing problems at the donor site despite the liposuction.

Some consider previous liposuction as a relative contraindication to the DIEP flap, however several case reports have demonstrated successful flap reconstruction [10]. In such cases, pre-operative imaging is vital to ensure several viable perforator options exist, such as by CTA [9]. While harvesting additional perforators may require more extensive dissection and could potentially increase donor-site morbidity and operative time, in this case, the decision reflected the operating surgeon’s preference after balancing flap perfusion with donor-site considerations. CTA is routinely used in our practice but was especially important in this case to assess the deep inferior epigastric artery and perforator anatomy, which could have been altered by prior surgery. Adequate flap perfusion can be further confirmed by intraoperative indocyanine green (ICG) laser angiography [11]. We did not need to use ICG as the entire flap was robustly perfused. There is significant variety in the nature and types of liposuction [12], and complication rates vary depending on individual risk factors [12]. There is limited literature on DIEP outcomes stratified by type of liposuction. As such, the decision on whether liposuction is a contraindication for DIEP flap often needs to be made empirically. The Stepwise Assessment and Full Evaluation in DIEP flap after abdominal liposuction (SAFE-DIEP) provides a framework for doing so, including CTA and colour doppler ultrasound pre-operatively, and ICG angiography intraoperatively [13].

The dissection was moderately challenging due to significant fibrosis from the prior abdominal liposuction and multiple abdominal scars. The total operative time was 11 h and 17 min, which was prolonged by the meticulous dissection required to preserve and include three perforators. Although this increased the operative time, we believed that maximizing flap perfusion contributed to a safe and problem-free reconstruction and a successful outcome

On the other hand, a study has shown that while the number of perforators may be marginally lower in patients with previous Pfannenstiel scars, the proportion of larger (>4mm) vessels is greater [14]. This may be due to ischaemic preconditioning of the flap because of division of the superficial inferior epigastric vessels during Caesarean section surgery. The larger vessels may mean improved perfusion thereby explaining the reduction in rates of partial flap loss reported in patients with prior abdominal surgical history [9].

The present case is the first to report a free DIEP flap around a pre-existing urostomy, although pedicled lower abdominal flaps have been used in gynaecological [15] and skin cancer [16] surgeries previously. These collectively show that urostomy may not be a complete contraindication to both pedicled and free DIEP flaps. We have further assessed the relative risks of several other surgeries on DIEP flap harvest and indicated possible concerns and implications for pre-operative planning (Table 2).

**Table 2.** Summary of previous abdominal procedure and relative risk for DIEP flap harvest as assessed by authors, principal concerns and implications for pre-operative planning.

Previous abdominal procedure	Relative risk for DIEP flap harvest	Principal concern	Implications for planning
Laparoscopic surgery	Low	Small scars from port incision may injure perforators.	Assess port scar location, unlikely to affect flap harvest. Skewing of flap to avoid scar if necessary [17].
Caesarean section	Low	Perforators are often larger in diameter, may aid perfusion [14,18].	Obtain CTA.
Open appendicectomy	Low - intermediate	Potentially unreliable vasculature at scar site.	Possible skewing of flap to avoid scar [17].
Open cholecystectomy	Low - intermediate	Open subcostal incisions may compromise abdominal skin perfusion.	Care taken to preserve a contralateral deep inferior epigastric perforator to maintain perfusion during closure [19].
Bowel surgery	Intermediate - high	Potential risk of donor site complications, fat necrosis [20].	Consider CTA, skewing of flap, counselling regarding increased risks.
Hernia repair	Intermediate - high	Mesh, fascial scarring, and perforator viability.	Review mesh position, consider CTA, concomitant repair, consider using lateral and/or multiple perforators [21].
Nephrectomy	Intermediate - high	Abdominal incisions may disrupt lateral perforators, weaken abdominal wall.	Assess operated side and consider contralateral flap harvest.
Urostomy	High	Stoma may disrupt perforators and fascia. Risk of parastomal hernia.	Obtain CTA and adjust flap boundaries appropriately. Few previously documented cases of successful DIEP flap in urostomy patients.
Open hysterectomy (with bilateral salpingo-oophorectomy)	High	Incisions may disrupt perforator viability and fascia.	Obtain CTA. Possibly consent for intra-operative decision to switch to pedicled transverse rectus abdominus myocutaneous flap [22].
Abdominal liposuction	High	Possible damage to deep epigastric vessels, venous drainage, reduction in fat volume [13].	Obtain CTA, consider bipedicled flap.

Several strategies were incorporated into this procedure to ensure its success, preparing multiple perforators identified on CT angiography and intraoperatively, avoidance of the urostomy when harvesting flap and ensuring perfusion of the anastomoses before final detachment of the contralateral hemi flap still left *in situ*. These are applicable to other donor sites as well, and the former two have been implemented in transverse rectus abdominis myocutaneous (TRAM) flaps as well [23].

## Conclusion

DIEP flap remains a viable option in patients with extensive abdominal and pelvic surgical history, even a urostomy, albeit with careful planning and execution. Further studies are needed to understand longer-term outcomes of such patients on a larger scale, especially patients with urostomies, for which there is a paucity of previous studies.

## Data Availability

All data generated in this study has been included in manuscripts and figures.
